# Comparative study on the chemical composition of laurel (*Laurus nobilis* L.) leaves from Greece and Georgia and the antibacterial activity of their essential oil

**DOI:** 10.1016/j.heliyon.2020.e05491

**Published:** 2020-12-21

**Authors:** Galina Stefanova, Tanya Girova, Velizar Gochev, Magdalena Stoyanova, Zhana Petkova, Albena Stoyanova, Valtcho D. Zheljazkov

**Affiliations:** a Lotos Expert OOD, 4000 Plovdiv, Bulgaria; b Department of Biochemistry and Microbiology and Department of Chemical Technology, Paisii Hilendarski University of Plovdiv, 24 Tzar Asen St., 4000 Plovdiv, Bulgaria; c Department of Analytical Chemistry and Physical Chemistry, Department of Tobacco, Sugar, Vegetable and Essential Oil Technology, University of Food Technologies, 26 Maritza Blvd., 4002 Plovdiv, Bulgaria; d Oregon State University, Department of Crop and Soil Science, 109 Crop Science Building, 3050 SW Campus Way, Corvallis, OR 97331, USA

**Keywords:** Laurel, Polyphenolsw, Essential oil, Chemistry, Natural product chemistry, Food science, Agricultural science, Biochemistry

## Abstract

Laurel (*Laurus nobilis* L.) is a plant species from Lauraceae family, and is native to the Mediterranean region. The goal of this study was to compare chemical composition of laurel leaves and antibacterial activity of its essential oil (EO) from wild-grown trees in Greece and Georgia. The laurel leaves from the two native habitats had dissimilar concentrations of phenolic acids. Of the conjugated flavonols and flavons, kaempferol (1981.3 μg/g) and apigenin (1433.6 μg/g) were the major representatives in the leaves from Greece, while luteolin (839.1 μg/g) and kaempferol (688.1 μg/g) were the major ones in the leaves from Georgia, respectively. The EO content was 1.42% and 4.54% in the leaves from Greece and Georgia, respectively. The main EO constituents of the Greek laurel plants were 1,8-cineole (30.8%), *α*-terpinyl acetate (14.9%), *α*-terpineol (8.0%), sabinene (7.9%), and terpinen-4-ol (6.0%). The main EO constituents of the Georgian laurel plants were 1,8-cineole (29.2%), *α*-terpinyl acetate (22.6%), sabinene (12.2%), and methyleugenol (8.1%). The EO antimicrobial activities against 20 microorganisms were determined. Among the Gram-positive bacteria, the *Enterococcus faecalis* strain was the most sensitive, followed by *Staphylococcus aureus* ATCC 6538. Among the *Candida* species, *C. albicans* ATCC 10231 was the most sensitive to the laurel leaf EOs.

## 1 Introduction

Laurel (*Laurus nobilis* L.) fam. Lauraceae is a shrub native to the Mediterranean region and cultivated in a number of countries in Asia, Europe, and the Americas as a spice, or used as ornamental plant (Parthasarathy et al., 2008). Laurel leaves, also known as bay leaves, are a major spice, which has been used traditionally as an ingredient for improving food flavor and taste. Naturally occurring biologically active compounds in leaves include terpenes, terpene derivates, polyphenols, alkaloids, minerals, vitamins (Caputo et al., 2017; Chahal et al., 2017; Parthasarathy et al., 2008).

The essential oil (EO) content of laurel leaves has been reported to range between 0.2% and 4.3% depending on the location, harvesting time, and EO extraction type and conditions (e.g. hydrodistillation or steam distillation) (Abu-Dahab et al., 2014; Bahmanzadegan et al., 2015; El et al., 2014; Fidan et al., 2019; Shokoohinna et al., 2014; Vasundhara et al., 2016). Previous research has shown that up to 270 EO constituents may be found in laurel leaves, the major ones being 1,8-cineole (22–56%), linalool (0.9–26.9%), *α*-terpinyl acetate (4.5–18.2%), *α-*pinene (2.2–15.9%), *β-*pinene (1.9–15.3%), sabinene (4.5–12.7%), *α*-terpineol (0.9–12.0%), terpineol-4 (0.9–4.1%) (Abu-Dahab et al., 2014; Bahmanzadegan et al., 2015; Chahal et al., 2017; El et al., 2014; Fidan et al., 2019; Goudjil et al., 2015; Shokoohinna et al., 2014; Vasundhara et al., 2016). Similarly to other EO-containing plants, the composition of laurel leaf EO has been shown to vary significantly as a function of the environment, genotype, and the type and duration of the distillation process (Abu-Dahab et al., 2014; Bahmanzadegan et al., 2015; Chahal et al., 2017; El et al., 2014; Fidan et al., 2019; Goudjil et al., 2015; Shokoohinna et al., 2014; Vasundhara et al., 2016). A recent retrospective on *L. nobilis* chemistry and biological activities of its EO was published by Chahal et al. (2017). In addition, laurel leaf EO has exhibited antimicrobial and antioxidant activities (Bahmanzadegan et al., 2015; Caputo et al., 2017; Dias et al., 2014; El et al., 2014; Fidan et al., 2019).

The main laurel leaf suppliers on the international market are Turkey, Portugal, Spain, and Iran, although native populations are rare and scattered around the Mediterranean, they could potentially be used for development of new varieties. Besides, wild-collected *L. nobilis* may have better nutritional value than cultivated laurel (Dias et al., 2014). The goal of this study was to compare the chemical composition of the laurel leaves from wild-grown trees found in two different European countries (Greece and Georgia) and assess the antimicrobial activity of their EOs against pathogenic and spoilage microorganisms.

## 2 Materials and methods

### 2.1 Chemicals

The following reagents and chemicals were used in this study: polyphenols, 1,8-cineole, and anhydrous sodium sulfate from Sigma-Aldrich; Nutritional Agar (NA); Sabouraud Dextrose Agar with chloramphenicol (SDA); Műller-Hinton Broth (MHB); Sabouraud Dextrose Broth (SDB); ciprofloxacin (CPH 5 μg/disc); fluconazole (FLC 25 μg/disc).

### 2.2 Plant material

Leaves from *L. nobilis* L. were collected only from wild growing trees, from one location per country. Subsamples were generated from 10 different trees per location by sampling leaves at different height and exposure within a tree. The leaves were harvested at the end of vegetation, during the formation of fruits. The leaves were collected in October 2016 from the Athos peninsula on a land belonging to the Bulgarian monastery (North Greece, at 160 m elev., 40°09′26″N and 24°19′35″E) and in December 2016 from the province of Meria (West Georgia, at 200 m elev., 41° 56′ 27″ N 41° 53′ 45″ E).

The collected samples were identified as *Laurus nobilis* L. by Dr. Ivanka Dimitrova at the University of Plovdiv “Paisii Hilendarski” in Plovdiv, Bulgaria. The moisture (39.7 ± 0.35% for leaves from Greece and 29.5 ± 0.35% for leaves from Georgia) was determined after drying at 105^o^С to constant weight (The State Pharmacopoeia of the USSR, 1990). The samples were analyzed for polyphenols and EO content and the values were presented on an absolute dry weight basis.

### 2.3 Determination of polyphenols

Sample preparation and HPLC analyses were carried out according to Marchev et al. (2011).

HPLC Analyses: Quantitative and qualitative measurements of phenolic acids and flavonoids were conducted using Waters 1525 Binary Pump HPLC systems (Waters, Milford, MA, USA) that had a Waters 2484 dual Absorbance Detector (Waters, Milford, MA, USA) and Supelco Discovery HS C18 column (5 μm, 25 cm × 4.6 mm) these operated under Breeze 3.30 software control.

Analyses of phenolic acids and flavonoids were carried out according to Ivanov et al. (2014) and Marchev et al. (2011).

### 2.4 Isolation of the essential oil (EO)

The EO was extracted via 3-h hydrodistillation of 100 g laurel leaves using Clevenger-type glass apparatus of the British Pharmacopoeia, modified by Balinova and Diakov (1974). The obtained EO was dried with anhydrous sodium sulfate, and placed in dark vials at 4 °C until gas chromatographic (GC) analysis.

#### 2.4.1 Gas chromatographic analyses for the chemical composition of essential oil

A GC analysis was carried out using an Agilent 7890A gas chromatograph, HP-5 column MS (30 m × 250 μm x 0.25 μm), temperature: 35 °C/3 min, 5 °C/min to 250 °C for 3 min, 49 min in total, helium as carrier gas, 1 ml/min constant speed, 30:1 split ratio. A gas chromatography–mass spectrometric (GC/MS) analysis was performed on an Agilent 5975C mass spectrometer, helium as carrier gas, column and temperature the same as in the GC analysis. The identification of the chemical constituents was made by comparison to their retention time and library data (Adams, 2007; NIST 08 database; own libraries). Components were listed according to their retention (Kovat's) indices, calculated using a standard calibration mixture of C_8_ - C_40_ n-alkanes in n-hexane. Compound concentration was computed as percentage of the total ion current (TIC).

### 2.5 Antimicrobial activity of EOs

The antimicrobial effects of the EOs were evaluated against Gram-positive bacteria *Bacillus cereus* ATCC 11778, *Enterococcus faecalis* (clinical isolate), *Enterococcus faecium* (clinical isolate), two strains of *Staphylococcus aureus* (ATCC 6538 and one food spoilage isolate), and *Listeria monocytogenes* NCTC 11994; Gram-negative bacteria: *Escherichia coli* ATCC 25922, *Klebsiella pneumoniae* (clinical isolate), two strains of *Salmonella abony* (ATCC 6017 and one clinical isolate), *Shigella flexneri* (clinical isolate), two strains of *Pseudomonas aeruginosa* (ATCC 27853 and one clinical isolate), and a food spoilage isolate of *Pseudomonas fluorescens*. *Proteus mirabilis* (clinical isolate), and *Proteus vulgaris* (clinical isolate); yeasts: two strains of *Candida albicans* (ATCC 10231 and one clinical isolate), *C. glabrata* (clinical isolate) and *C. tropicalis* (clinical isolate). The clinical isolates from bacteria were kindly provided by the Department of Microbiology and Immunology at the Medical University of Plovdiv, and the yeast isolates were kindly provided by the National Referent Laboratory of Mycology at the National Center of Infectious and Parasitic Diseases in Sofia, Bulgaria.

All strains were deposited in the Microbial Culture Collection of the Department of Biochemistry and Microbiology at Paisii Hilendarski University of Plovdiv Bulgaria.

The antibacterial activity of the EOs was assessed according to the Clinical Laboratory Standard Institute (CLSI) reference method for antimicrobial disk susceptibility tests (CLSI, M2-A9 2006; CLS, M7-A7 2006). The anticandidal activity of the EOs was assessed according to the CLSI reference method for antifungal disk diffusion susceptibility testing of yeast (CLSI, M27-A3 2008; CLSI, M44-A2 2009). The used filter paper discs (Whatman №1) in disc diffusion test were prepared by soaking with 10 μL of the tested essential oil samples. 1,8-Cineole represents about 30% of the total oil content and the filter paper discs were prepared by soaking with 3 μL of the 1,8-cineole. The used positive controls of reference antibiotic discs Ciprofloxacin (5 μg/disc) and antimycotic Fluconazole (25 μg/disc) were purchased by HiMedia (India). The antimicrobial activity of the EOs and 1,8-cineole determined by disc diffusion tests was expressed as inhibitory zone (IZ) diameter in mm; these zones were measured to the nearest millimeter using an antibiotic zone scale. The antimicrobial activities of CPH and FLC were also determined as positive controls.

### 2.6 Statistical analysis

All experiments were performed at least three times. All data were presented as mean ± standard error of the mean. Statistical significance was assessed by either Student's-test or one-way analysis of variance (ANOVA) followed by Tukey's multiple comparison Differences between means were considered statistically significant if p > 0.05.

## 3 Results and discussion

### 3.1 Polyphenol composition

The polyphenol content is shown in Table 1.

**Table 1:** Polyphenol content of laurel leaves from Greece and Georgia (mean ± SD).

№	Compounds	RT, min	Content, μg/g dw			
			Leaves from Greece		Leaves from Georgia	
			Free	Conjugated	Free	Conjugated
Hydroxycinnamic acid derivates						
1.	Rosmarinic acid	6.54	47.5 ± 0.08	-a	-	-
2.	Chlorogenic acid	33.13	243.9 ± 0.36	-	-	48.1 ± 0.04
3.	Caffeic acid	38.59	586.1 ± 0.67	56.8 ± 0.09	31.4 ± 0.05	789.3 ± 0.81
4.	*р*-Coumaric acid	46.59	151.1 ± 0.12	246.6 ± 0.35	45.3 ± 0.06	375.9 ± 0.36
5.	Sinapic acid	47.14	607.7 ± 0.72	560.4 ± 0.52	-	1513.9 ± 1.22
6.	Ferulic acid	47.69	300.1 ± 0.41	2193.0 ± 2.11	29.4 ± 0.04	70.4 ± 0.09
7.	Cinnamic acid	53.41	135.0 ± 0.11	486.2 ± 0.51	22.7 ± 0.04	513.4 ± 0.71

Hydroxybenzoic acid derivates						
8.	Gallic acid	9.47	24.8 ± 0.03	-	-	-
9.	Protocatechuic acid	19.19	-	17.2 ± 0.02	13.3 ± 0.02	68.6 ± 0.07
10.	Salicylic acid	28.87	207.3 ± 0.36	41.7 ± 0.04	-	29.4 ± 0.03
11.	Vanillic acid	37.84	-	83.9 ± 0.12	37.0 ± 0.04	-
12.	Syringic acid	39.86	242.0 ± 0.21	19.6 ± 0.01	5.9 ± 0.0	789.1 ± 0.72

Flavonols						
13.	Myricetin	15.63	124.5 ± 0.11	75.2 ± 0.08	75.5 ± 0.08	47.2 ± 0.04
14.	Quercetin	21.46	48.9 ± 0.05	42.3 ± 0.04	65.3 ± 0.07	44.9 ± 0.05
15.	Kaempferol	26.78	122.2 ± 0.11	1981.3 ± 2.00	250.7 ± 0.24	688.1 ± 0.70

Flavons						
16.	Luteolin	23.89	4.8 ± 0.0	388.6 ± 0.36	59.0 ± 0.06	839.1 ± 0.84
17.	Apigenin	27.93	268.6 ± 0.26	1433.6 ± 1.12	161.7 ± 0.15	262.7 ± 0.25

Flavanon						
18.	Hesperetin	16.09	-	116.4 ± 0.11	-	31.2 ± 0.03

Quercetin glycosides						
19.	Rutin	7.42	217.4 ± 0.20	-	-	-
20.	Hyperoside	8.98	141.8 ± 0.13	-	-	-

a Compound not found.

In this study, twelve phenolic acids were quantified in the laurel leaves (Table 1). Overall, the results showed significant differences in the free and conjugated phenolic acid content, which was determinated after acid hydrolysis. In the laurel leaves from Greece, in the hydroxycinnamic acid derivates, sinapic (607.7 μg/g), caffeic (586.1 μg/g), and ferulic (300.1 μg/g) acids were the major free phenolic acids, while ferulic (2193.0 μg/g), sinapic (560.4 μg/g), and cinnamic (486.2 μg/g) acids were the major conjugated acids. The major free hydroxybenzoic acid derivates were syringic (242.0 μg/g) and salicylic (207.3 μg/g) acids, while vanillic acid (83.9 μg/g) was the major conjugated acid. Kaempferol (1981.3 μg/g) and apigenin (1433.6 μg/g) were the major representatives of the group of conjugated flavonols and flavons, respectively. In the group of quercetin glycosides only the free form of rutin and hyperoside in the Greek leaves was identified. In the laurel leaves from Georgia, *p*-coumaric acid (45.3 μg/g) was the major free phenolic acid in the group of the hydroxycinnamic acid derivates, while sinapic (1513.9 μg/g), caffeic (789.3 μg/g), and cinnamic (513.4 μg/g) acids were the major conjugated acids. In the group of the hydroxybenzoic acid derivates the dominnat was syringic acid (789.1 μg/g). Luteolin (839.1 μg/g) and kaempferol (688.1 μg/g) were the major constituents of the group of conjugated flavonols and flavons, respectively. The derivates of the group of conjugated cinnamic acids were the dominant compounds in the Georgian leaves, while the level of flavonols and flavons was lower (Table 1).

The observed differences in the polyphenol composition of the samples from the two countries can be explained by the different environmental conditions at the two collection sites. Mount Athos on the Athos peninsula (Greece) is on the Aegean coast, and the village of Meria in Western Georgia is near the Black Sea. The long-term average annual precipitation at the sampling site in Greece (Hilandar on Mount Athos) is 474 mm. At that location, the average temperature in July is 25 °C, while the one in January is 8 °C. Also, most of the precipitation occurs in December, with an average of 80 mm of rain. On the other hand, the climate at the sampling location in Western Georgia, around the village of Meria, is warm, humid, mild, and temperate. The weather station at Ozurgeti, Georgia (around 10 km from the collection site at Meria) has long-term average annual precipitation of 1981 mm, with average temperature in August of 22.7 °C, and average temperature inf January of 5.0 °C. In general, the results reported here showed that climatic differences in both regions caused significant differences in leaf polyphenols. Therefore, our results are in agreement with previous literature reports on other plants, which are cultivated in different geographic regions (Dragovic-Uzelac et al., 2007; Franquet-Griell et al., 2012).

### 3.2 Essential oil yield and composition

The EOs from the two locations were light yellow and had a specific odor.

The EO content of the Greek laurel leaves was 1.42 ± 0.01% (v/w), whereas the EO content of the Georgian laurel leaves was 4.54 ± 0.04% (v/w). The EO content of laurel leaves was similar to that in some literature reports but different from other reports, most probably due to environmental and genotypic differences. Previous studies reported that the EO content of dried laurel leaves was 0.2% to 4.3% (Abu-Dahab et al., 2014; Bahmanzadegan et al., 2015; El et al., 2014; Fidan et al., 2019; Shokoohinna et al., 2014; Vasundhara et al., 2016). The reported differences in the EO content and yield between this study and literature reports may also be due to environmental, harvest, and postharvest processing factors.

The chemical composition of the EOs of laurel leaves from Greece and Georgia is shown in Table 2.

**Table 2:** Composition of the essential oil of laurel leaves from wild-grown trees in Greece and Georgia (mean ± SD).

№	Components		RT, min	RI^a^	Content, (% of TIC^b^)	
					Leaves from Greece	Leaves from Georgia
1.	*α-*Thujene	MH	8.95	931	0.3 ± 0.0	0.7 ± 0.0
2.	*α-*Pinene	MH	9.22	939	5.3 ± 0.04	5.5 ± 0.05
3.	Camphene	MH	9.71	954	0.6 ± 0.0	0.2 ± 0.0
4.	Sabinene	MH	10.58	971	7.9 ± 0.07	12.2 ± 0.09
5.	*β*-Pinene	MH	10.72	979	3.6 ± 0.05	3.7 ± 0.03
6.	*β*-Myrcene	MH	11.05	991	0.5 ± 0.0	1.3 ± 0.02
7.	*α*-Phellandrene	MH	11.96	1003	0.3 ± 0.0	0.8 ± 0.0
8.	1,8-Cineole	OM	12.50	1032	30.8 ± 0.29	29.2 ± 0.25
9.	*γ*-Terpinene	MH	13.28	1055	3.3 ± 0.04	0.6 ± 0.0
10.	Linalool	OM	14.63	1096	-c	3.8 ± 0.03
11.	Terpinen-4-ol	OM	17.09	1179	6.0 ± 0.05	1.8 ± 0.03
12.	*α*-Terpineol	OM	17.56	1189	8.0 ± 0.10	1.7 ± 0.03
13.	Bornyl acetate	OM	20.01	1269	1.2 ± 0.02	-
14.	*α*-Terpinyl acetate	OM	21.87	1333	14.9 ± 0.13	22.6 ± 0.20
15.	Eugenol	PP	21.98	1363	2.7 ± 0.04	0.8 ± 0.0
16.	Methyleugenol	PP	23.17	1371	3.6 ± 0.05	8.1 ± 0.08
17.	*β*-Elemene	SH	24.20	1390	-	0.1 ± 0.0
18.	*β*-Caryophyllene	SH	24.49	1429	0.4 ± 0.0	0.4 ± 0.0
19.	Germacrene D	SH	25.18	1484	0.3 ± 0.0	0.1 ± 0.0
20.	Elemicin	SH	26.10	1522	-	1.1 ± 0.0
21.	Caryophyllene oxide	OS	27.49	1574	1.8 ± 0.03	0.2 ± 0.0
22.	Spathulenol	OS	27.61	1619	0.4 ± 0.0	0.2 ± 0.0
23.	*β*-Eudesmol	OS	28.05	1642	-	0.7 ± 0.0
24.	n-Heptadecane	AH	29.27	1700	0.2 ± 0.0	0.1 ± 0.0
25.	n-Heneicosane	AH	32.50	2100	0.6 ± 0.0	0.2 ± 0.0
26.	Phytol	D	32.86	2105	1.5 ± 0.02	0.1 ± 0.0
27.	n-Docosane	AH	33.79	2200	0.7 ± 0.0	0.2 ± 0.0
28.	n-Tricosane	AH	35.00	2300	0.4 ± 0.0	0.1 ± 0.0
29.	n-Tetracosane	AH	36.13	2400	0.3 ± 0.0	0.1 ± 0.0
30.	n-Pentacosane	AH	38.10	2500	0.5 ± 0.0	0.2 ± 0.0
31.	n-Hexacosane	AH	39.88	2600	0.8 ± 0.0	0.3 ± 0.0
32.	n-Heptacosane	AH	41.72	2700	0.9 ± 0.01	0.3 ± 0.0
33.	n-Octacosane	AH	44.40	2800	0.3 ± 0.0	0.1 ± 0.0
34.	Squalene	T	45.02	2817	0.9 ± 0.01	0.3 ± 0.0
Aliphatic hydrocarbons (AH),%					4.79	1.66
Monoterpene hydrocarbons (MH),%					22.10	25.50
Oxygenated monoterpene (OM),%					61.46	60.40
Sesquiterpene hydrocarbons (SH),%					0.73	1.71
Oxygenated sesquiterpenes (OS),%					2.18	0.39
Diterpenes (D),%					1.49	0.11
Triterpenes (T),%					0.90	0.30
Phenyl propanoids (PP),%					6.37	9.93

a RI – retention (Kovat's) index.

b TIC – total ion current; All data are presented as mean value ± standard deviation (n = 3).

c nd – below 0.05% of TIC or not detected.

Overall, 30 EO constituents, or 99.0% of the total oil content were found in the Greek laurel EO (Table 2). Fourteen of the EO constituents were with concentrations above 1%. The main EO constituents were: 1,8-cineole (30.8%), *α-*terpinyl acetate (14.9%), *α-*terpineol (8.0%), sabinene (7.9%), terpinen-4-ol (6.0%), *α*-pinene (5.3%), *β*-pinene (3.6%), methyleugenol (3.6%), and *γ*-terpinene (3.3%).

Thirty-three constituents, or 97.8% of the total oil were identified in the Georgian laurel EO. Eleven of these EO constituents were in concentrations over 1%. The major ones in the Georgian laurel EO were: 1,8-cineole (29.2%), *α-*terpinyl acetate (22.6%), sabinene (12.2%), methyleugenol (8.1%), *α-*pinene (5.5%), linalool (3.7%), and *β-*pinene (3.7%). Some of the differences in the chemical composition of the laurel leaf EOs in this study and in some reports may be due to environmental and genetic factors.

Overall, the composition of the Greek and Georgian laurel EOs in this study varied within the range of the laurel leaf chemical composition reported previously. Previous reports showed that the laurel leaf EOs produced from different regions was rich in 1,8-cineole (5.7–6,3%), *α-*terpinyl acetate (5.9–25.7%), linalool (3.7–16.4%), sabinene (2.3–14.1%), methyleugenol (3.1–12.5%), terpinen-4-ol (3.4–11.1%), limonene (5.3%), *α-*pinene (3.00–7.4%), and *β-*pinene (1.6–13.4%) (Abu-Dahab et al., 2014; Bahmanzadegan et al., 2015; El et al., 2014; Fidan et al., 2019; Goudjil et al., 2015; Shokoohinna et al., 2014; Vasundhara et al., 2016).

Oxygenated monoterpenes (1,8-cineole, *α*-terpinyl acetate, *α*-terpineol, terpinen-4-ol) were the dominant group in the Greek laurel EO, followed by monoterpene hydrocarbons (sabinene, *α*-pinene, *β*-pinene, *γ*-terpinene), phenyl propanoids (methyleugenol) and aliphatic hydrocarbons. Oxygenated monoterpenes were also the dominant group in the Georgian laurel EO, followed by monoterpene hydrocarbons and phenyl propanoids.

### 3.3 Antimicrobial activity of the essential oils (EOs)

In this study, the laurel leaf EO samples demonstrated antimicrobial activity against all of the Gram-positive bacteria and yeast tested (Table 3).

**Table 3:** Antimicrobial activity of laurel leaf essential oils from wild-grown trees in Greece and Georgia (mean ± SD).

Test microorganisms	Inhibitory zone diameter (IZ), mm			Antibiotics
	Laurel leaf essential oil from Greece 10 μl/disc	Laurel leaf essential oil from Georgia 10 μl/disc	1,8-cineole 3 μl/disc	Ciprofloxacin
Gram-positive bacteria				
*B. cereus* ATCC 11778	18.0 ± 0.18	12.0 ± 0.13	10.2 ± 0.30	28.0 ± 0.27
*E. faecalis* (clinical isolate)	52.0 ± 0.53	11.5 ± 0.13	10.0 ± 0.11	26.0 ± 0.26
*E. faecium* (clinical isolate)	20.0 ± 0.19	12.0 ± 0.13	10.0 ± 0.11	ni
*S. aureus* ATCC 6538	40.0 ± 0.38	12.5 ± 0.13	10.5 ± 0.13	30.0 ± 0.29
*S. aureus* (clinical isolate)	19.0 ± 0.19	15.0 ± 0.16	12.5 ± 0.13	30.0 ± 0.29
*L. monocytogenes* NCTC 11994	12.0 ± 0.13	10.0 ± 0.10	9.5 ± 0.11	18.0 ± 0.17

Gram-negative bacteria				
*E. coli* ATCC 25922	15.6 ± 0.17	ni^a^	8.4 ± 0.08	21.5 ± 0.20
*K. pneumoniae* (clinical isolate)	10.0 ± 0.11	ni	ni	13.0 ± 0.13
*S. abony* ATCC 6017	14.0 ± 0.14	9.5 ± 0.11	8.4 ± 0.08	12.5 ± 0.13
*S. abony* (clinical isolate)	14.0 ± 0.14	9.0 ± 0.10	8.4 ± 0.08	18.5 ± 0.20
*S. flexneri* (clinical isolate)	14.0 ± 0.14	ni	ni	16.5 ± 0.14
*P. aeruginosa* ATCC 27853	ni	ni	ni	15.0 ± 0.14
*P. aeruginosa* (clinical isolate)	ni	ni	ni	12.0 ± 0.13
*P. fluorescens* (clinical isolate)	ni	ni	ni	16.8 ± 0.17
*P. mirabilis* (clinical isolate)	11.0 ± 0.12	ni	ni	12.0 ± 0.13
*P. vulgaris* (clinical isolate)	12.0 ± 0.13	8.5 ± 0.08	8.2 ± 0.08	12.5 ± 0.13

Yeasts				Fluconazole
*C. albicans* ATCC 10231	24.8 ± 0.24	16.3 ± 0.14	14.2 ± 0.13	21.5 ± 0.21
*C. albicans* (clinical isolate)	21.0 ± 0.21	16.0 ± 0.14	14.1 ± 0.11	22.5 ± 0.22
*C. glabrata* (clinical isolate)	15.0 ± 0.14	10.0 ± 0.10	9.1 ± 0.11	21.0 ± 0.20
*C. tropicalis* (clinical isolate)	16.6 ± 0.15	12.0 ± 0.13	10.1 ± 0.11	16.5 ± 0.16

a ni – no inhibition detected.

Among the Gram-positive bacteria, the strain belonging to *E. faecalis* was the most sensitive, followed by *S. aureus* ATCC 6538, whereas *L. monocytogenes* NCTC 11994 was the most resistant. Among the species belonging to the *Candida* genus, *C. albicans* was the most sensitive and *C. glabrata* was the most resistant to the tested laurel leaf EOs. Also, *P. aeruginosa* and *P. fluorescens* were resistant to the tested EO samples. *S. flexneri* and *K. pneumoniae* were resistant to the EOs. Overall, the Gram-positive bacteria and yeasts were more sensitive to the tested EOs in comparison with the Gram-negative bacteria, which is in agreement previous reports (Griffin et al., 1999).

In general, the laurel leaf EO from Georgia demonstrated weaker inhibitory activity. The difference in the antimicrobial activities of the tested EO samples was most probably due to the differences in their chemical composition. The content of oxygenated monoterpenes in the two oils was different; e.g. 1,8 cineole, *α-*terpinyl acetate, *α*-terpineol, terpinen-4 ol, *etc.,* and the presence of these compounds may explain the antimicrobial activity of EOs (Griffin et al., 1999). Antimicrobial activity of 1,8-cineole, which is the most abundand component of both laurel EOs was also studied. Pure 1,8-cineole demonstrated weaker antimicrobial activity in comparison with EOs, which means that antimicrobial activity of laurel EOs could not be attributed only to the dominant compound but the additional effect of some minor compounds as well as synergistic effects may have played a role.

The antimicrobial activity of the tested EOs in this study was comparable to the activity of the antibacterial antibiotic ciprofloxacin and antimycotic fluconazole with the exception of the EO activity against *Pseudomonas* spp. The different antimicrobial resistance of both types of bacteria was probably due to the different structure and chemical composition of the cell wall of Gram-positive and Gram-negative bacteria. Indeed, the so-called “*external membrane*”, which is typical for Gram-negative bacteria, could prevent or delay the diffusion of the EO extract from the nutritive medium through the cell wall and membrane into the cytoplasm.

The EOs also demonstrated antimicrobial activity against three species of medically important yeasts belonging to the *Candida* genus. Overall, NAC species *C. glabrata* and *C. tropicalis* were more resistant in comparison with *C. albicans*.

The results from this study confirmed published data on the antimicrobial activities of laurel EOs (El et al., 2014; Goudjil et al., 2015). However, in some of the previous reports, the laurel leaf EO was isolated following different methods, and the plant material originated from other locations with different environmental conditions.

The results from this research were utilized by members of the author's team for the development of documentation needed for steam distillation processing equipment and facility of laurel leaves from wild trees around the Bulgarian monastery in Mount Athos, Greece. Consequently, in 2019, laurel leaves EO was isolated at this processing facility. The resulting EO is currently being evaluated for its utilization in various food, cosmetic and other consumer products, subject to further research by the research team. In addition, members of the research team are developing documentation for the industrial processing of the wild grown laurel around the village of Meria, Georgia.

## 4 Conclusions

This study found significant dissimilarities in the chemical profile of laurel leaf EOs from Greece and Georgia. Derivates of hydroxycinnamic and hydroxybenzoic acids, flavonols and flavons dominated in the laurel leaves from Greece, whereas the dominant compounds in the Georgian leaves were the group of conjugated cinnamic acids. The main group of constituents in the EOs from Greek and Georgian laurel leaves was composed of the oxygen monoterpenes 1,8-cineole, *α-*terpinyl acetate, *α*-terpineol, and terpinen-4-ol. The EOs demonstrated antimicrobial activity against pathogenic and spoilage microorganisms. Laurel plants from Greece and Georgia show promise as a new source of laurel leaves for the international spice market.

## Declarations

### Author contribution statement

Galina Stefanova, Tanya Girova, Velizar Gochev, Magdalena Stoyanova, Zhana Petkova, Valtcho D. Zheljazkov: Performed the experiments; Analyzed and interpreted the data; Wrote the paper.

Albena Stoyanova: Conceived and designed the experiments; Performed the experiments; Analyzed and interpreted the data; Wrote the paper.

### Funding statement

This research did not receive any specific grants from funding agencies in the public, commercial, or not-for-profit organization. APC was paid by startup funds of Oregon State University awarded to Dr. Valtcho D. Jeliazkov (Zheljazkov).

### Data Availability Statement

Data included in article.

### Declaration of interests statement

The authors declare no conflict of interest.

### Additional information

No additional information is available for this paper.
